# Autism and heritable bone fragility: A true association?

**DOI:** 10.1016/j.bonr.2018.04.002

**Published:** 2018-04-18

**Authors:** Meena Balasubramanian, Rebecca Jones, Elizabeth Milne, Charlotte Marshall, Paul Arundel, Kath Smith, Nicholas J. Bishop

**Affiliations:** a Highly Specialised Severe, Complex & Atypical OI Service, Sheffield Children's NHS Foundation Trust, UK; b Sheffield Clinical Genetics Service, Sheffield Children's NHS Foundation Trust, UK; c Academic Unit of Child Health, University of Sheffield, UK; d Department of Psychology, Sheffield Children's NHS Foundation Trust, UK; e Department of Psychology, University of Sheffield, UK; f Medical School, University of Sheffield, UK; g Sheffield Diagnostic Genetics Service, Sheffield Children's NHS Foundation Trust, UK

**Keywords:** Autism, Osteogenesis imperfecta, Bone fragility, Autism assessments, Genomic studies

## Abstract

**Objectives:**

Osteogenesis Imperfecta (OI) is a heterogeneous condition mainly characterised by bone fragility; intelligence is reported to be normal. However, a minority of children seen also show symptomology consistent with an ‘Autism Spectrum Disorder’. A joint genetics and psychology research study was undertaken to identify these patients using ‘Gold Standard’ research tools: Autism Diagnostic Inventory Revised (ADI-R); Autism Diagnostic Observation Schedule (ADOS) and undertake genetic analyses in them.

**Method:**

A cohort of *n* = 7 children with autistic traits and severe/complex OI were recruited to the study. The study was set-up to explore whether there was a genetic link between bone fragility and autism in a sub-set of patients with bone fragility identified with autism traits in our complex/severe OI clinic. This was not set-up as a prevalence study but rather an exploration of genetics in association with ADI/ADOS confirmed ASD and bone fragility.

**ADI& ADOS:**

Standardised tools were used to confirm autism diagnosis. ADI and ADOS were completed by the Clinical Psychologist; ADI comprises a 93 item semi-structured clinical review with a diagnostic algorithm diagnosing Autism; ADOS is a semi-structured assessment of socialisation, communication and play/imagination which also provides a diagnostic algorithm.

**Exome sequencing:**

In patients recruited, those that fulfilled research criteria for diagnosis of autism using above tools were recruited to trio whole exome sequencing (WES).

**Results:**

one patient had compound heterozygous variants in *NBAS*; one patient had a variant in *NRX1*; one patient had a maternally inherited *PLS3* variant; all the other patients in this cohort had pathogenic variants in *COL1A1/COL1A2*.

**Conclusions:**

Although, not set out as an objective, we were able to establish that identifying autism had important clinical and social benefits for patients and their families in ensuring access to services, appropriate schooling, increased understanding of behaviour and support.

**Lay summary:**

It is important for clinicians looking after children with brittle bone disease, also referred to as Osteogenesis Imperfecta (OI) to be aware of early features of developmental delay/autistic traits especially with severe forms of OI as the emphasis is on their mobility and bone health. Ensuring appropriate assessment and access to services early-on will enable these patients to achieve their potential. Further investigations of genomics in bone fragility in relation to autism are required and dual diagnosis is essential for high quality clinical and educational provision.

## Highlights

• Osteogenesis Imperfecta is the commonest form of heritable bone fragility disorder with an incidence of 1 in 15,000 live births
• Intelligence is usually reported to be normal; however, this study describes association of autistic traits with OI
• It is important to undertake autism assessments early in case of clinical suspicion of ASD as children with OI would benefit from early educational intervention
• Early identification and clarification of diagnosis of ASD in children with OI will ensure that children are able to achieve their full potential.

## 1 Introduction

Osteogenesis imperfecta is a heterogeneous group of disorders characterised by bone fragility and fractures. Extra-skeletal features such as hearing loss, dentinogenesis imperfecta and joint hypermobility can also be variably present. The condition can be inherited in an autosomal dominant or recessive pattern, or can be caused by a sporadic mutation (*de novo*) in a proband (Balasubramanian M. Clinical and Molecular Heterogeneity of Osteogenesis Imperfecta, 2017). Osteogenesis imperfecta is the most common form of inherited bone fragility disorder, with an estimated prevalence of 1 in 15,000 live births (Folkestad et al., 2017). Incidence is approximately 1/15,000–1/20,000 live births but this may be underestimated, as milder forms may not have come to medical attention (Forlino and Marini, 2016).

The classification of this disorder was traditionally based on severity and inheritance. Previously, the four main types of osteogenesis imperfecta have been separated into the following distinct categories (Sillence et al., 1979). A greater understanding of genetics has led to an extension of the classification of OI. Over 85% of mutations causing OI are in the type 1 collagen genes (*COL1A1* or *COL1A2*); the most common being the replacement of a glycine amino acid in the (Gly – X – Y)_n_ repeating unit within the collagen triple helix. Apart from the type 1 collagen gene, many other genes are now confirmed to be associated with OI. Recurrent mutations in *IFITM5* have been implicated in the aetiology of Type V OI, which has an AD pattern of inheritance (Semler et al., 2012; Cho et al., 2012).

OI Types VI-IX are inherited in an autosomal recessive (AR) pattern (Gensure et al., 2005; Glorieux et al., 2002; Glorieux, 2005). Other genes such as, *CRTAP*, *P3H1, FKBP10*, *PPIB*, *SP7*/Osterix (*OSX*), *SERPINF1*, *SERPINH1*, are associated with AR forms of OI (Alanay et al., 2010; Baldridge et al., 2008; Christiansen et al., 2010; Lapunzina et al., 2010; van Dijk et al., 2009). These forms are typically very severe if not lethal. More recently several other recessive forms of OI- *TMEM38B/BMP1/CREB3L1/SPARC* have been characterised (Shaheen et al., 2012; Martínez-Glez et al., 2012; Symoens et al., 2013; Mendoza-Londono et al., 2015) and X-linked forms of OI (*PLS3/MBTPS2*) (van Dijk et al., 2009; Lindert et al., 2016) and heterozygous variants in *WNT1/LRP5* (Laine et al., 2012; Hartikka et al., 2005) making OI a very genetically heterogeneous condition and perhaps use of heritable bone fragility as a more appropriate terminology to describe this group of conditions.

Rarely patients may present who do not fit into the sub-categories of this extended OI classification. This may be because they have not yet suffered a fracture, or because they present with other pathologies, such as the syndromal features of facial dysmorphism, craniosynostosis or contractures. They may have extreme short stature or developmental delay. In these cases, it may be that the patient has an atypical diagnosis of a type I collagenopathy (Balasubramanian et al., 2016). Some patients with bone fragility display autistic traits which are not in keeping with their clinical diagnosis as children with OI are reported to have normal intelligence; this would be classified as ‘atypical bone fragility’.

In the UK, the prevalence of autism is 1 in 100 (Baird et al., 2006). Over the last 5 years, in our centre which has a large cohort of bone fragility patients, it was our clinical observation that an unexpectedly high number of children with bone fragility are also presenting with clinical traits of ASD as characterised in DSM V (2013) (Autism Spectrum Disorder 299.00 (F84.0) DSM-V, American Psychiatric Association, 2013) (*n* = 10–15/102). We observed that the rate of affected children appeared to be higher than expected from the latest ASD population prevalence estimates of 1.9% (Baird et al., 2006) and decided to study this in further detail. There is sparse evidence for this association in the literature but in our clinical practice we have noted a clear association, which seems more pronounced in children with relatively severe bone fragility.

The DSM V diagnostic criteria for ASD specify a child or adult must show a) persistent deficits in social communication and social interaction, b) restricted and repetitive patterns of behaviour, c) symptoms must be present in early developmental period, d) symptoms must cause impairment in functioning and e) symptoms must not be better explained by developmental delay. This study explored the association between bone fragility and autism spectrum disorder in further detail and set out to describe a novel phenotypic association.

## 2 Materials and methods

The research involved participation of children with OI from the nationally-commissioned Severe and Complex OI group (total 102 children). Participants were recruited into a research project to study the association of autism and OI and establish genotype: phenotype correlations. Funding was obtained from the Newlife Charity and ethical approval was obtained from the local regional ethics committee (REC reference: 15/YH/0196) to undertake phenotyping and genetic work-up in this group of patients.

From this group, we selected patients aged between 3 and 16 years (total of 10 patients), who were reported to have difficulties with social interaction by the multi-professional team. The Senior Clinical Psychologist assessed these patients clinically for those who show signs of ASD (*n* = 10 children were noted to have atypical social skills, 7 of these families were approached; all of them consented to participate in the study). 3/10 patients: it was decided by the clinical team to not approach children in whom atypical social skills were noted as it was felt by the multidisciplinary team that the families would not be able to deal with a diagnosis of autism in addition to the severe bone fragility. Following informed consent from parents and their carers and where applicable, assent from children, eligible children were recruited to the study. This was not set out as a prevalence of autism in bone fragility study (planned as a next step) but it is likely from our observation that may be as high as 10% in our cohort.

Recruited children were screened for ASD traits using standardised ASD clinical research tools. Sub-group of children who screened positive for ASD underwent a dysmorphology assessment and genetic testing to identify common genotypes within this sub-group. We discussed results with families and onward referral to local child development centres in those that fulfilled diagnostic criteria. This optimised follow-up and support for families within ASD services locally along with continued support within OI services.

### 2.1 ASD screening

Recruited children were screened by the Senior Clinical Psychologist using the Autism Diagnostic Inventory – Revised (ADI-R) and Autism Diagnostic Observation Schedule (ADOS).

#### 2.1.1 Standardised psychological tests

##### 2.1.1.1 ADI-R (Autism Diagnostic Inventory Revised) (Le Couteur et al., 2008)

The ADI-R is a clinical diagnostic instrument for assessing autism in children and adults. It provides a diagnostic algorithm consistent with DSM-IV and ICD-10 criteria for ASD. It consists of a 93-item semi-structured interview for parents/carers of people with suspected ASD. The ADI-R scores are categorised into three domains of communication and language, social interaction and restricted/repetitive behaviours. A classification of ASD is given when scores in all three domains meet specified cut-offs. The assessment can be conducted from 4 years of age to adult.

##### 2.1.1.2 ADOS (Autism Diagnostic Observation Schedule) (Lord et al., 1997)

The ADOS is a semi-structured assessment and observation of socialisation, communication and restricted/repetitive behaviours. The ADOS is completed by the clinician directly with the child and includes various activities designed to elicit behaviours that are coded to inform an ASD diagnosis. Sub-sections are coded using an algorithm; children score in the categories of Non-spectrum, Autism or Autism-Spectrum. The assessment can be conducted from 12-months of age to adult.

From this group, children identified as having ASD using ADI-R and ADOS were included for genetic assessment.

### 2.2 Genetic assessment

From the children recruited, those that screened positive for a diagnosis of ASD/Autism using the ADI-R and ADOS screening tools, were invited for a genetic assessment by the Consultant Clinical Geneticist. These patients underwent assessments including detailed family pedigree and dysmorphology examination to ascertain if there are other genetic reasons for the ASD (*i.e.* dual diagnoses). Following appropriate consent, clinical photographs were taken and blood samples obtained for genetic analysis. The Clinical Geneticist assessed these patients and performed a comprehensive dysmorphology examination to ascertain whether ASD is part of a “syndromal” diagnosis or whether there are other contributory factors within the family and/or medical history.

### 2.3 Genetic analyses

Samples were analysed from those that screened positive on ASD assessments for 60 K microarrays which is a detailed analysis of chromosomes and next-generation sequencing of genes implicated in ASD/Autism (autism/developmental delay gene panel). Common variants were identified from within this cohort of patients.

## 3 Clinical report

In total seven patients were recruited to the study and underwent autism and genetics assessments. We did not approach all the families identified by the clinical team where this was not considered to be appropriate at that point in time (*n* = 3). Below are detailed summaries of patients recruited and assessment outcomes.

### 3.1 Patient 1

Patient 1 was an 11-year old boy, the second child of non-consanguineous parents. There was no family history of bone fragility or autism. The pregnancy was normal, and the patient was delivered by caesarean section post term after failure of labour progression. He was treated in the Special Care Baby Unit for two days after delivery due to pyrexia. He was born with left-sided calcaneus talipes equinovarus and right-sided developmental dysplasia of the hip. His undescended testes were operated on successfully.

Patient 1 is developmentally delayed. He walked at 2.5 years of age and had delayed onset of speech. He had difficulties with fine motor skills and attended a school for children with special needs. This patient had a clinical diagnosis of ASD made at 5-years of age. His parents also reported ritualistic behaviours, resulting in a referral to Child and Adolescent Mental Health Services for an assessment of possible obsessive compulsive disorder.

He was noted to have previously suffered finger fractures and a decrease in vertebral height. A DXA scan to measure his bone mineral density (BMD) undertaken before commencement of bisphosphonate treatment demonstrated a reduced BMD with *Z*-scores of −3.4 at the lumbar vertebrae and a total body score of −2.5 when adjusted for age and gender. A bone biopsy had demonstrated low turnover trabecular osteopenia consistent with osteoporosis.

Also of note, he had diagnoses of asthma and idiopathic generalised epilepsy. He suffered from intermittent neutropenia thought to be the result of sodium valproate therapy. He received 3-monthly pamidronate infusions, remained on melatonin daily and had been prescribed midazolam, to be given in the event of a prolonged seizure.

On examination, he had bilateral low-set ears, blue sclerae and glasses due to hyperopia.

Trio whole exome sequencing (WES) in him identified a *de novo* missense variant in *NRXN1* which is known to be associated with neurodevelopmental disorders/autism and being further investigated as it is known to interact with *COL1A1*.

### 3.2 Patient 2

Patient 2 was an 11-year old boy, the second child to healthy, non-consanguineous parents. There is no family history of bone fragility and autism. He was born in the breech position spontaneously at 32-weeks gestation after the pregnancy was complicated by placental abruption, causing severe abdominal pain and heavy bleeding. At birth, he weighed 1.76 kg (9th centile); he required continuous positive airway pressure for 24 h and phototherapy to treat his neonatal jaundice. He was fed *via* a nasogastric tube for the first week of life.

He failed to thrive throughout childhood with height and weight below the 0.4th centile and head circumference 0.4th-2nd centile, with insufficient weight gain resulting in the insertion of a percutaneous gastrostomy for nutritional support. He suffered frequent infections including bronchiolitis, pneumonia and urinary tract infections. A micturating cystourethrogram identified bilateral vesicoureteric reflux. He had consistent hypogammaglobulinaemia and lymphopenia throughout childhood with poor vaccine responses. This patient received 3-weekly immunoglobulin replacement therapy. Also of note, he had bilateral optic atrophy and consistently abnormal liver function tests.

Patient 2 had severe intellectual disability. He had delayed speech and suffers from gross and fine motor delay: he first walked at 19 months. He demonstrated significant echolalia and restricted interests; the patient had received a clinical diagnosis of ASD at 6-years of age.

He had suffered several fractures of the vertebrae, metatarsals and tibias. A bone biopsy at 7-years of age demonstrated a high rate of bone turnover and osteopenia, with marked subperiosteal bone resorption. DXA scans showed reduced bone mineral density, however it was difficult to determine the degree of reduction due to his small size. He received 3-monthly pamidronate infusions.

The patient had undergone numerous investigations throughout his life to provide an explanation for his clinical features. Trio WES identified that patient 2 is compound heterozygous for c.3010C>T and c.5741G>A pathogenic mutations in the *NBAS* gene (Balasubramanian et al., 2017). He had been diagnosed with SOPH syndrome (Short Stature, Optic Atrophy, Pelger-Huet anomaly), which largely explains the patient's clinical picture. On examination, he had short stature and high pitched voice. Facial dysmorphism included a prominent forehead, low set ears, hypertelorism, proptosis, progeric appearance to his skin and up-slanted palpebral fissures.

### 3.3 Patient 3

Patient 3 was a 4-year old boy, the third child of healthy, non-consanguineous parents. There was no family history of bone fragility or autism. Bowing of the lower limbs observed on the anomaly scan raised antenatal suspicion of a campomelic dysplasia. The patient was born by normal vaginal delivery at term. He weighed 3.74 kg (50th centile) with a head circumference of 34 cm (25th centile). He suffered mild respiratory distress at birth but did not require ventilatory support.

A skeletal survey performed after birth demonstrated a normal thoracic cage volume, bowing of the long bones with abnormal metaphyses and a fractured ulna. The patient suffered fractures to his left humerus and right forearm. He was subsequently diagnosed with severe osteogenesis imperfecta.

By 4-years of age, he had suffered multiple fractures of his ulnas and humeri, a femoral fracture and multiple vertebral wedge fractures. He has undergone bilateral osteotomies and rodding of his femurs and tibias at 2 and 3 years of age, respectively. He received 3-monthly pamidronate infusions.

This patient was developmentally delayed, sat independently from 2 years and walked with aids from 2.5 years of age. He had delayed speech and required intervention from speech and language therapists at age 21 months. He has demonstrated “rocking” behaviour from 2.5 years of age but did not have a clinical diagnosis of ASD before recruitment to the study.

On genetic assessment, he was noted to have a ‘triangular’ face, blue sclerae, high-pitched voice in keeping with a diagnosis of ‘Classical OI’. He went on to have testing for *COL1A1/A2* and was found to have a pathogenic c.902G>A variant in *COL1A2*. This pathogenic mutation is predicted to replace glycine at position 301 with a glutamic acid. Glycine substitutions are well-recognised as a cause of OI. This confirmed his clinical diagnosis of OI.

### 3.4 Patient 4

Patient 4 was a 14-year old male, the only child born to non-consanguineous parents. His younger half-brother (through same mother) had learning difficulties but there was no other family history of autism. The pregnancy was normal with delivery by caesarean section at 39 weeks due to a breech presentation. He had a birth weight of 3.54 kg (65th centile). He needed oxygen shortly after delivery but was not admitted to the Special Care Baby Unit. He had global developmental delay: no head control was evident at 4 months; sitting was achieved at 2 years of age; the patient walked at 4.5 years and currently uses a wheelchair. He spoke his first words aged 7 years. He was doubly incontinent and has learning difficulties; he attended a school for children with special needs. He was diagnosed with ASD at 5-years of age, before recruitment to the study after demonstrating little eye contact and having restricted interests. He had previously engaged in self-harm behaviour such as head banging and biting.

Patient 4 had suffered from a fractured forearm and vertebral wedge fractures. He had been given a diagnosis of probable primary osteoporosis, suffering discomfort in his back and lower limbs. DXA scanning undertaken before commencing 3-monthly pamidronate infusions demonstrated a reduced BMD when adjusted for age and gender of −2.6 at lumbar vertebrae 2–4 and a total body measurement of −2.7. He had joint hypermobility and brittle nails.

This patient was diagnosed with bilateral femoral proximal anteversion, which was operated on with a derotation osteotomy. He demonstrated ligamentous laxity and suffered a leg length discrepancy. The patient had a small scrotum and incomplete descended testes. He also had left sided choroidal coloboma and myopia.

On examination, he was not dysmorphic. So far WES in him has not identified any variants of significance and further genetic analysis is ongoing.

### 3.5 Patient 5

Patient 5 was a 13-year old male, the first child to healthy, non-consanguineous parents. There was a family history of osteoporosis in his maternal grandfather but no family history of autism. The pregnancy was not planned and was not detected until approximately 25 weeks. No scans were performed. He was born at term and was immediately well after birth.

His initial development was normal, with gross motor milestones being achieved as expected: he sat up aged 6 months and walked at age 8 months. His speech was delayed; he started speaking at 5 years of age after receiving speech therapy. He was diagnosed with ASD at 3-years of age after concerns were raised at his toddler group. The patient attended a school for children with special needs.

He had suffered three fractures: two of his forearm and one of his wrists. Additionally, he had suffered from multiple crush fractures of his thoracic and lumbar vertebrae. The small joints of the fingers were hypermobile, but there was little evidence of hypermobility elsewhere. DXA scans undertaken before commencing bisphosphonate treatment demonstrated reduced BMD, with *Z*-scores of −2.7 at the lumbar vertebrae and − 2.6 total body measurement when adjusted for age and gender. He had a diagnosis of idiopathic osteoporosis with a bone biopsy at 12-years of age demonstrating severe low turnover cortical and trabecular osteopenia. The patient received 3-monthly infusions of pamidronate.

On examination, this patient was not dysmorphic. WES identified a maternally inherited *PLS3* pathogenic variant which explained his bone fragility.

### 3.6 Patient 6

Patient 6 was an eight year old boy, the second child of healthy, non-consanguineous parents. There was no family history of bone fragility or autism. Shortened long bones were identified on the 16-week scan and the child was delivered by caesarean section at 37-weeks. At birth, he needed ventilation with a bag and mask. He was born with fractures of all the long bones and multiple ribs: he was diagnosed with severe OI antenatally. The patient was treated in the special care baby unit for three months; he was fed *via* a nasogastric tube and suffered from gastroesophageal reflux.

He developed a right sided inguinal hernia shortly after birth which was surgically corrected at one month of age. He also suffered from fusion between the base of his skull and top of his spinal column. Throughout his life, he had suffered multiple long bone fractures, including several femoral fractures and fractures of his radii. He had undergone several surgical procedures, with bilateral femoral and tibial rodding procedures undertaken at 4 and 5 years of age, respectively. His bone fragility was managed with 3-monthly infusions of pamidronate.

He was developmentally delayed: he started talking between two and a half and three years of age and started to “commando crawl” at 3 years of age. He had never walked. The patient attended a mainstream school after starting a year later than his peers. He did not have a previous diagnosis of ASD.

On examination, he had short stature, blue sclerae, triangular face and dentinogenesis imperfecta. There were marked deformities of his long bones, resulting in a pes cavus appearance. Genetic testing showed that he carried a *de novo* pathogenic variant in *COL1A1* c.2282G>A in exon 33/34 confirming his clinical diagnosis of OI.

### 3.7 Patient 7

Patient 7 was a 6-year old boy, second child of healthy, non-consanguineous parents with no significant family history. His sister was said to have a seizure disorder of unknown aetiology but there was no family history of autism. Antenatally, there were concerns with short long bones and bowed femur and he was born at term with a normal birth weight. He was noted to have multiple fractures and commenced on treatment with pamidronate with a good response. He was noted by the therapy team to have autistic traits and recruited to the study. He fulfilled the criteria for a diagnosis of autism. On examination, he had a triangular face, blueish sclerae, high-pitched voice, dentinogenesis imperfecta, significant limb deformities and scoliosis. Genetics analyses revealed normal microarrays and a pathogenic variant was identified in *COL1A2* confirming his clinical diagnosis of OI. c.2533G>A mutation in exon 37 of *COL1A2* gene, this pathogenic mutation is predicted to replace glycine at position 845 with an arginine and has previously been reported in individuals with OI confirming his diagnosis.

## 4 Molecular analysis

### 4.1 Patient 1

Unclassified *de novo* missense variant in *NRXN1* being further investigated.

### 4.2 Patient 2

Compound heterozygous for *NBAS* variants; maternally inherited c.3010C>T variant and paternally inherited c.5741G>A variant (Balasubramanian et al., 2017).

### 4.3 Patient 3

*De novo* pathogenic c.902G>A variant in *COL1A2.*

### 4.4 Patient 4

No causative variant identified so far on exome sequencing.

### 4.5 Patient 5

Hemizygous for a maternally inherited, c.1295T>A pathogenic mutation in exon 12 of *PLS3*.

### 4.6 Patient 6

*De novo* pathogenic variant in *COL1A1* c.2282G>A in exon 33/34 confirming his clinical diagnosis.

### 4.7 Patient 7

*De novo* pathogenic c.2533G>A mutation in exon 37 variant was identified in *COL1A2* confirming his clinical diagnosis. Table 1 provides a detailed overview of recruited patients and corrsponding genotype.

**Table 1:** Description of clinical phenotype and genotype in autism and bone fragility cohort.

	Patient 1	Patient 2	Patient 3	Patient 4	Patient 5	Patient 6	Patient 7
Age	11-years	11-years	4-years	14-years	13-years	8-years	6-years
Family History	NA	NA	NA	ID in half-brother	Osteoporosis in maternal grandfather	NA	Seizure disorder in sister
Fractures	++	++	++	++	+	++	+++
Pamidronate	Yes	Yes	Yes	Yes	Yes	Yes	Yes
ID	Moderate-severe	Moderate-Severe	Moderate	Moderate-severe	Mild	Severe	Severe
ASD	Yes	Yes	Yes	Yes	Yes	Yes	Yes
Molecular analysis	*NRXN1* c.1273T>A *de novo* missense variant	*NBAS* c.3010C>T and c.5741G>A variants	*COL1A2* c.902G>A *de novo* variant	No causal variants so far on WES	*PLS3* c.1295T>A mat variant	*COL1A1* c.2282G>A *de novo* variant	*COL1A2* c.2533G>A *de novo* variant

ID: Intellectual disability; ASD: Autism spectrum disorder; NA: Not applicable.

## 5 Discussion

Children with OI/heritable bone fragility are said to have normal intellectual development and the emphasis is usually on the motor delay (Balasubramanian et al., 2017). Autism spectrum disorder (ASD) is not a proven association and is often overlooked in this group. This results in delayed diagnosis and input, thereby leading to reduced impact of early intervention within this group.

Diagnosis of ASD takes considerable time and may not occur in the early years of a child's life (Zwaigenbaum et al., 2013). Zwaigenbaum et al., 2013 in a recent review concluded that early diagnosis of ASD enhances the impact of interventions and reduces parental burden. Families experience significant stress and uncertainty during this period and interventions designed to reduce symptoms and improve functioning are delayed due to late interventions. This delay is further compounded in children with bone fragility due to their disability potentially masking ASD symptoms. We have observed an increased incidence of ASD within our OI cohort (*n* = 10 out of 102) based on general population estimates of 1 in 100 children (Baird et al., 2006).

ASD and heritable bone fragility are not known to be associated disorders. There is emerging evidence to suggest that patients with autism spectrum disorders (ASD) are at an increased risk for fractures and peri-pubertal boys with ASD have lower BMD than age-matched controls (Ekhlaspour et al., 2016). A study performed by Neumeyer et al., 2013 evidenced a lower bone mineral (BMD) density in boys with ASD than controls. It has been found that children with ASD are at an increased predisposition to a fracture of any kind than those without the disorder (Furlano et al., 2014) and people below the age of 50 with ASD are at increased risk of suffering a hip fracture than controls (Neumeyer et al., 2015).

It has so far been thought any association between bone fragility and ASD is environmental in origin, due to a diet insufficient in vitamin D or lack of physical activity (Neumeyer et al., 2013) and there is no literary evidence of a syndromal condition consisting of genetic bone fragility and ASD. More recently, studies by Neumeyer et al., 2017a, Neumeyer et al., 2017b have very well demonstrated that bone accrual and bone microarchitecture is impaired in ASD with reduction in bone strength and resultant low BMD. This may yet be attributable to low levels of physical activity and calcium intake in children with ASD but it is also plausible that there may be as yet un-identified genetic modifiers that play a role in this predisposition. The study described here comes from the opposite angle: identifying patients with severe bone fragility and pointing out the increased incidence of ASD in this cohort which would be worth investigating further in a larger cohort of patients.

This study undertook detailed clinical and molecular phenotyping in a cohort of children presenting with autistic traits and bone fragility; to determine if this is a novel phenotype and whether they share a common genetic aetiology; and add clinical definition to the association of ASD and OI. A similar example would be the identification of an association between OI and a profound neurological phenotype caused due to mutations in *WNT1* (Fahiminiya et al., 2013; Faqeih et al., 2013). This has led to further studies demonstrating the effect of WNT-ß Catenin signaling pathway on cellular differentiation in both the skeleton and the central nervous system (Palomo et al., 2014; Tang, 2014). Identification of similar findings in this condition would be only be possible by a thorough dysmorphic assessment and genetic analyses in order to establish common phenotypic and genotypic characteristics.

In our cohort, we identified diverse genotypes as opposed to a single genetic aetiology which reflects the genetic heterogeneity of both ASD and bone fragility. Type 1 collagen variants were the most common which reflects the most common cause of OI; *NRXN1* is known to be associated with ASD and it would be interesting to explore how this links with the bone fragility. *NBAS* is known to cause SOPH syndrome and *PLS3* are known to be associated with heritable bone fragility but autism is not a known association with these genes. It is likely that similar to high risk candidate genes in ASD (https://www.sfari.org/resource/sfari-gene/), there is going to be diverse genotypes in this group. Further studies to explore this association and undertake deep sequencing in this cohort may well identify other candidate genes and interacting biological pathways that may be relevant.

Although as detailed in the ‘Results’ section above, we have not been able to identify a common molecular aetiology for patients presenting with bone fragility and ASD, we have been able to determine that patients with bone fragility, especially those at the severe end of the spectrum/presentation with additional clinical features, seem to have a higher incidence of ASD. Being able to undertake detailed autism assessments in this group has allowed earlier diagnosis in some of the younger patients within this group. The numbers studied here are small and there is a planned national study across more centres which may help define this association further.

As expected from the literature, the majority of patients recruited to the study have variants in *COL1A1/A2*, the commonest genes associated with OI. It would be interesting to ascertain through a national study with larger patients' number whether there are additional risk factors early on in life that contribute to an increased incidence of autistic traits in patients presenting with severe bone fragility and type 1 collagen variants.

## 6 Summary

By exploring the genetic causality of autism in bone fragility, we are essentially identifying a genetic link between autism and childhood bone fragility. Osteogenesis Imperfecta which is the commonest inherited form of bone fragility does not usually present with intellectual disability and/or autism and hence, proving this association will be crucial in informing early diagnoses and prognostic information for families with this rare bone disease. From the study undertaken in seven families so far, we have been able to show that early diagnosis impacts educational support and allocation of additional resources for children who already have very complex medical needs.

Therefore, it is important for clinicians treating children with OI to be aware of this potential association, have a high index of suspicion when children display autistic traits, and to refer for an autism assessment and formal diagnosis so as to ensure adequate support is provided early on in a child's development. It requires a shift in focus of children with bone fragility care to not just be targeted at their fracture management and motor development but also focused on their intellectual development. With better treatments for bone fragility becoming available, it is important that these children are adequately supported so they can reach their full potential not only in terms of their physical health but also their mental wellbeing. It is also important that further studies are undertaken to explore this association and draw firmer conclusion on this associated phenotypes.
